# Establishing a community advisory group (CAG) for partnership defined quality (PDQ) towards improving primary health care in a peri-urban setting in KwaZulu-Natal, South Africa

**DOI:** 10.1186/s12913-020-05275-6

**Published:** 2020-05-08

**Authors:** Thoko Ndaba, Myra Taylor, Musawenkosi Mabaso

**Affiliations:** 1 Discipline of Public Health, School of Nursing and Public Health Medicine, University of KwaZulu-Natal, Durban, South Africa; 2 Human and Social Capabilities (HSC) Research Division, Human Sciences Research Council, Durban, South Africa

**Keywords:** Community, Health facility, Partnership, Maternal, Child, Neonate, Health

## Abstract

**Background:**

Community advisory groups (CAGs) have been shown to be catalysts who bridge the gap between communities and primary health care facilities by sustaining good working relationships through community engagement to improve the quality of the health care services. This study aimed to explore the establishment, operation, and accomplishments of a CAG towards building a strong partnership between the health facilities and local communities in support of the Partner Defined Quality (PDQ) process, to improve the delivery of quality maternal and neonatal care in a peri-urban setting in the province of KwaZulu-Natal, South Africa.

**Methods:**

The study used a qualitative exploratory research design. Recruitment followed a purposive sampling approach. The study targeted leadership representatives from the community, potential beneficiaries, and health care providers in the selected catchment areas. Participants were identified during community mobilization events that took place during the preparatory stage to ensure key stakeholder support. A participatory research approach was used to discuss membership, composition, the selection criteria, including formulation, and agreement on terms of reference of the CAG membership, roles and responsibilities. A rapid assessment method was used for data collection and analysis of establishment of the CAG, its activities and accomplishments.

**Results:**

The community nominated 24 CAG members during the consultative meetings and the organogram provides clear terms of reference, roles and responsibilities. Immediately after inception, the CAG used four indicators (weaknesses, threats and risks, strengths, and opportunities) to review the community and primary health care challenges that affect their communities. These CAG activities were linked with the phases of the PDQ process. The CAG committed itself going forward to continue to create an enabling environment for all stakeholders working to improve the well-being of the community, especially the PDQ teams working on improving the care of pregnant mothers and their babies pre- and post-delivery.

**Conclusion:**

This work shows that developing community relationships and infrastructure are critical initial stages before embarking on PDQ planning and implementation. Empowerment, local ownership, funding, technical resources and ongoing support are critical elements for sustainability of CAG activities.

## Background

The South African maternal mortality ratios (MMRs) declined between 2009 and 2015 from 189 per 100, 000 live births to 138 per 100, 000. However, MMR remains high [1]. In addition, the national neonatal mortality rate (NMR) also remains high and accounted for 44% of the infant mortality rate (27 per 1, 000 live births), and 32% of the under-5 mortality rate (37 per 1, 000 live births) in 2015 [2]. KwaZulu-Natal province has one of the highest maternal and neonatal mortality [3]. The challenges are multifactorial and also include poor health status and care of women, illiteracy and lack of information with regard to available health services, poor antenatal and obstetric care both within the community and at health facilities, absence of a well-trained cadre of health extension workers, inadequate referral systems and /poor linkages of health centres with the communities [1, 4].

Consequently, the South Africa government re-committed itself to address these challenges as part of the new Sustainable Development Goals (SDGs), through evidence-based health packages providing universal coverage for maternal, neonatal and child interventions [5]. This involves strengthening the continuum of care linking home, community, primary health care, regional and district hospitals by ensuring the availability of the right care in the right place at the right time at each level [6, 7]. Integrated health care for maternal and newborn health has been adopted for national application at different levels of the health system, including family and community, outpatient and maternal units and, district and regional hospitals [7].

Pivotal to the successful implementation of community interventions for the reduction of maternal, newborn, and child death, is the establishment and maintenance of stakeholder partnership strategies to ensure sustainability of the bi-directional continuum of care from facility to community level [8, 9]. However, part of the challenge of bridging the gap between communities and primary health care facilities is strengthening the partnership process, which includes community entry and mobilization, building and maintaining partnerships, and engagement towards improved community health outcomes [6, 7]. Community advisory groups (CAGs) have been shown to be catalysts who bridge the gap between communities and primary health care facilities by sustaining good working relationships through community engagement to improve the quality of the health care services [10].

The CAG serves as a community advisory body whose main aim is to promote the development of a mutually beneficial and meaningful partnership between health researchers and community stakeholders [10]. This approach is used to make communities fully understand the risks and benefits in order to protect them from exploitation and harm [10–12]. CAGs provide a public forum for community members to present, engage and discuss issues of interest or concerns that affect them as a community [10, 13]. CAGs work with existing groups within communities, which are made up of representatives of diverse community interests. However, CAGs vary in terms of composition, representation and selection processes. Models of CAGs vary depending on the population under study, terms of reference, roles and responsibilities [10, 14].

Consequently, in this study a CAG was established was initiated as a first step to prepare for the implementation of the Partnership Defined Quality (PDQ) in order to build a strong partnership between health facilities and local communities. The PDQ is a partnership model that seeks to mobilise and advocate for quality health care services for communities [15]. It assists communities and health workers in finding solutions to problems in quality care delivery and access. It works on improving interaction, trust, and accountability through a shared vision of responsibility between communities and service providers. It advocates for stronger partnerships to enhance delivery of quality health care to bridge the gap to achieve better health outcomes [15].

This study explored the establishment, operation, and accomplishments of a CAG towards building a strong partnership between the health facilities and local communities in support of the PDQ process in order to improve the delivery of quality maternal and neonatal care in a peri-urban setting in KwaZulu-Natal, South Africa.

## Methods

### Study setting

The Umlazi Township is located approximately 17 km South of Durban’s Central Business District [16]. The area of Umlazi is 4, 481.7 ha and forms part of eThekwini Municipality in KwaZulu-Natal. The population of Umlazi is approximately one million residents. The area is characterised by severe housing shortages, major informal settlements, high levels of unemployment and little economic development [16]. The health challenges include high HIV prevalence rates and tuberculosis (TB), poor case finding of HIV and TB exposed children, late antenatal care (ANC) booking, and low contraceptive uptake, resulting in unplanned pregnancies and high neonatal mortality [17].

The study area has 12 Department of Health (DOH) Primary Health care (PHC) centres and five Municipal clinics. Three of the 12 DOH clinics open each day for 24 h. These PHCs have a very high workload: the average catchment population per clinic greater than 60, 000 people [17]. Catchment areas of the 12 DOH facilities were selected for the establishment of CAG. The idea was to select PHC facilities with a high workload, as this affectes the utilization rate and quality of the services.

### Study design and procedure

The study used a qualitative exploratory research design. A purposive sampling approach was used to draw the target group from communities in the selected catchment areas. The study targeted leadership representatives from the community, potential beneficiaries, and health care providers. The target population for the study was wide and inclusive to ensure a holistic and community centred approach. Participants were identified during community mobilisation events that took place during the preparatory stage to ensure key stakeholder support.

A participatory research approach was used to discuss the size of the membership, participation, composition and the selection criteria, and to formulate and agree on the terms of reference, and discuss the CAG roles and responsibilities. A rapid assessment method was used for the data collection and analysis of the CAG activities and achievements [18]. This involved a series of consultative meetings and training workshops on the structure, terms of reference as well as the proposed roles and responsibilities of the CAG. Permission to record and document CAG workshops, activities and meetings for synthesis and analysis of data was obtained in the first regular CAG meeting.

### Stakeholder consultation

In consultation with the local government councillors and health facilities in the study areas, the first CAG information session meeting was held with a few key community stakeholders involved in TB and HIV activities in the community. A plan to establish a CAG focussing on the care of mothers and their newborn babies in the community was discussed. Following an agreement, dates, venues and invitations were sent to other key community stakeholders. The planned meetings involved Ward Councillors, health personnel from Primary Health Care facilities (PHCs), pastors, traditional healers, business and general community members. The main aim of the second meeting was to:

- Inform and educate community stakeholders about CAG
- Discuss membership size, participation, composition and the selection criteria
- Formulate and agree on terms of reference (TOR)
- Discuss CAG roles and responsibilities

The third consultative meeting covered the discussion of current issues. The review of action items was undertaken through question and answer sessions. This led to the development of a mission statement, specific purpose, scope, goal and specific objectives. This was followed by the formation of a CAG through a nomination process and a show of hands. Selected members were tasked with the responsibility of scouting for more GAG members, specifically of persons of good standing in the community. The fourth consultative meeting was a summit that involved the formal introduction of the CAG to the community, the establishment of the CAG agenda, and advocating for a wider community support for the establishment of partnerships with clinics.

### Data synthesis and analysis

A rapid assessment approach was used for the data collection, synthesis and analysis of the establishment of the CAG, and its activities and accomplishments. This is an exploratory methodology designed to provide an understanding of a situation based on the integration of data, including the process by which events and actions take place. The data were collected within a short period, without a formal examination of transcribed or coded data [18]. This type of qualitative inquiry was especially suited to our research questions because our study focused on the process of establishment of the CAG and the interaction between CAG members, health facility staff and the community.

The data collection concentrated on the documentation of the notes gathered during CAG training workshops, priority-setting sessions, and consultative meetings with the community and the health facility staff. This was supplemented by observation of the CAG activities and the minutes generated during their regular meetings. These documents were carefully read and analysed to understand the CAG activities, challenges and opportunities identified in bridging the gap between the communities and the PHC facilities towards supporting the implementation PDQ process. In order to improve quality of services the PDQ process requires four stages. These are:

- Phase 1 (planning, designing and building support)
- Phase 2 (exploring quality)
- Phase 3 (bridging the gap)
- Phase 4 (working in partnerships)

## Results

### CAG organogram

Table 1 shows the composition of the CAG. In addition to the general community members, the CAG included people from health facilities, faith organizations, and a non-profit organization. An important component were members of Sukuma Sakhe, which is a provincial programme that was founded on the premise of taking government to the people in a coordinated manner. “Sukuma Sakhe” is actually a Zulu phrase, which means stand up and build.

**Table 1:** Composition of community advisory group (CAG) for partner defined quality (PDQ) in an urban setting in KwaZulu-Natal, South Africa

Executive CAG Members	Number
Chairman	1
Deputy chair	1
Secretary	1
Deputy secretary	1
Other members	
Professional nurses	3
Clinic committee member	1
Teachers	2
CCGs	7
Sukuma Sakhe members	2
Additional community members	5

### CAG terms of reference

It was agreed that the chairperson would always conduct meetings in an open transparent manner, encouraging open constructive engagements. It was also agreed that the chairperson would always strive to achieve consensus among CAG members. The terms agreed upon were as follows:

- The CAG members will try to attend all meetings scheduled and will always be on time.
- If a member was not going to be able to attend a meeting he/she will forward an apology to the chair or secretary prior to the date of the meeting.
- It was emphasised that all meetings should be as participatory as possible to create an enabling environment to address community issues.
- It was suggested that CAG members should per invitation avail themselves if invited by other stakeholder to meetings that deal with community issues to strengthen partnerships.
- The CAG members were to participate in creating continuous dialogue and collective action for improved quality of health services
- The CAG members were requested and encouraged to share information and learn from each other so as to promote support and strengthen PHC.
- Informing and educating the community about the importance of PDQ
- The CAG was to continuously monitor and review its own work together with the PDQ quality teams.
- It was also agreed that urgent matters would not wait to be tabled at monthly meetings, but would be discussed immediately by the available members and sometimes together with partners.
- The chairperson and vice chairperson were also to review all action items from the previous meeting and their outcomes and keep a record for future reference.
- Record keeping was highlighted as one of the best practices that should be adhered at all times.

### CAG activities

Immediately after the its inception of the CAG chose four indicators to review the Community and Primary Health Care challenges that affect their communities. The indicators included probing (1) weaknesses, (2) threats and risks, (3) strengths, and (4) opportunities. These activities were linked with the third and fourth phase of the PDQ process. The CAG members divided themselves into commissions to work on these indicators. After weeks of interactions between the commissions, community and health facilities in the areas following feedback was given:

### Weakness

- Poor attitude of PHC staff towards clients especially, in communication and health care delivery
- Time of arrival to start work at PHC and time of departure is sometimes not honoured
- In some PHC facilities there are no covered waiting spaces and privacy especially for chronic TB and HIV patients collecting their test results and treatment
- The cleanliness in some of these PHC facilities is very poor and the shortage of general cleaning staff makes it difficult to maintain hygiene

### Threats and risks

- Some PHC facilities have inadequate security for staff during both days and nights
- Some PHC facilities have no transport provision for undertaking community outreach programmes
- Where transport was available there was no security for health personnel and government cars when doing community outreach even when visiting crime hot spots

### Strengths

- Besides sometimes experiencing a shortage of resources these PHC centres mostly serve clients beyond their catchment areas.
- Clients come from different areas, sections and wards and some come from outside the province.
- Operating managers are available to maintain operations and relationships with clients in spite of the overcrowding.
- Communities noted successful immunization campaigns provided by the PHC facilities in spite of many challenges.
- Communities also noted youth campaigns run by PHCs to empower youth on health issues.

### Opportunities

- It was suggested that the newly established CAG was in a better position to address PHC-related weaknesses, threats and risk through PDQ health teams.
- There were opportunities to train and empower community caregivers (CCGs) on mother, neonatal and child health for care of mother and baby pre-and post-delivery as part of the PDQ processes.
- It was also suggested that the CAG, working with the community and other stakeholders such as community policing forums, was in a better position to address the social ills identified in the community.

Primary Health Care personnel acknowledged that patients complain at most visits, and mentioned that they were aware that the waiting periods are unacceptably long due to shortage of manpower, as posts at the time were not filled. One of the PHC professional said, “*We request cooperation and harmony from the members of the community so as to work in partnership for the smooth running of our service.*” Another PHC professional also responded by saying “*We pledge to strengthen communication between CAG, community members, CCGs and other stakeholders by conducting inclusive monthly meetings to tackle, discuss and solve together challenges that plague our quality service delivery to our community*.”

After the report back session was completed, the chairperson of the CAG said, “*It looks like there is much to tackle and it is clear that it will be very important that we work together in partnership”*.

### Way forward

The CAG committed itself going forward to continue to create an enabling environment for all stakeholders working to improve the well-being of the community, especially PDQ teams. They pledged:

- to encourage mothers in the community to attend antenatal care and avoid home deliveries.
- to monitor quality improvements at PHC facilities as part of the PDQ process through quality improvement teams.
- to securing grants or other funding to sustain the work of the CAG, and that this should be an ongoing activity.
  - Towards this end the CAG initiated the first self-sustenance or income-generating project (a vegetable garden and a poultry farm) with a view of expanding these initiatives across the community.
- to continue even amid funding difficulties by identifying other means to promote member retention and ensuring that the benefits of membership outweigh the costs through exposure to training opportunities, access to information and resources, and public recognition such as that provided in local media.

## Discussion

The effort of establishing a CAG needs community mobilisation, time, commitment and patience. It is not easy if there is a climate of community instability ranging from claims of poor service delivery in the area to violence. It is advisable to step back for some time until the community settles and people have time to concentrate and engage. This also involves many consultative meetings with gate keepers, key community stakeholders, and general community members to ensure the actual establishment of the CAG.

The successful establishment of the CAG was facilitated by working closely with the “Sukuma Sakhe” programme, which had been launched in all the 11 districts in the province of Kwa-Zulu Natal, South Africa. The aim of the programme is to have communities working together to confront problems that plague communities such as TB, HIV/AIDS, maternal and neonatal morbidities and mortalities, crime, abuse of women and children to name a few key concerns [19]. The ward councillor chairs the meeting and the members are from its community. They have their meetings on a monthly basis to review progress. The importance of a CAG and its activities was discussed in these meetings. During this process all participants were allowed to ask questions, voice out concerns if any, and offer suggestions where necessary. Regular communication with communities has been shown to enable community-based organizations to gain local support and to improve the quality of community health programmes [20, 21].

Consequently, the CAG has turned out to be the eyes and ears on the ground for community leaders to report any social ills that would affect the community negatively. This was facilitated by the participatory approach adopted in all CAG activities which has been observed to allow for direct uptake and utilisation of PHC facilities [20–22]. Through this approach the CAG has also turned out to be the driver of early detection of abnormal signs and symptoms on neonates and advocate for early referral to PHC facilities. It now partners and works with CCGs on the ground to facilitate immediate care for mothers and their babies at a community level. They assist in calling ambulances if mothers go into labour to get them to the PHC as soon as possible. The CAG in this community continues to be an integral part of efforts to strengthen communication, partnerships and quality delivery of health care services through the PDQ process.

However, while the focus of establishing CAG was meant to support the implementation of the PDQ, other community priorities also emerged and took over much of the agenda during CAG meetings. These included violent crime, drugs and gender-based violence. The community leaders highlighted the need for a demand-driven agenda / approach to ensure relevance. Another challenge for the CAG was ensuring sufficient penetration and reach across the target community.

### Limitations

This study has some limitations. Rapid assessment as a data collection and analysis methodology is mostly carried out while in the field without a formal, systematic examination of transcribed and coded data. It is designed to capture a multi-layered perspective of an event, system or process based on the integration of data collected within a short period. The CAG was implemented in one administrative ward and thus the findings cannot be generalised to the other districts or provinces, which may have different context. However, the primary goal of establishing a CAG for building a stronger partnership between the local communities and selected PHC facilities in order to support of the PDQ process improved delivery of quality maternal and neonatal care was realised. This paper makes a contribution to the literature on the role of CAGs in building strong partnerships with communities to improve quality of care. As this study shows, CAGs are a useful approach to engaging communities in the process of improving the quality of local PHC services.

## Conclusion

This work shows that developing community relationships and infrastructure are critical initial stages before embarking on programme planning and implementation, and they require continued nurturing if the conditions for community participation are to be sustained. However, sustainability may be enhanced by drawing on existing community resources such as “Sukuma Sakhe” in this instance. Equally important is ensuring empowerment and local ownership of the CAG. This requires sufficient funding, technical resources, and ongoing support of community efforts.

## Data Availability

Data sharing is not applicable to this article as no datasets were generated or analysed during the current study.

## Abbreviations

ANC: Antenatal Care
AIDS: Acquired immunodeficiency syndrome
BREC: Biomedical Research Ethics Committee
CHS: College Of Health Sciences
CAG: Community advisory group
CCG’s: Community care givers
DOH: Department of Health
HIV: Human immunodeficiency virus
MMR: Maternal mortality rate
NMR: Neonatal mortality rate
PDQ: Partnership defined quality
PHC: Primary Health Centre
SDG: Sustainable development goals
TB: Tuberculosis
